# The Gut Microbiome in Multiple Sclerosis: A Potential Therapeutic Avenue

**DOI:** 10.3390/medsci6030069

**Published:** 2018-08-24

**Authors:** Trevor O. Kirby, Javier Ochoa-Repáraz

**Affiliations:** Department of Biology, Eastern Washington University, 258 Science Building, Cheney, WA 99004, USA; trevor.kirby@eagles.ewu.edu

**Keywords:** multiple sclerosis, microbiome, therapeutics, experimental autoimmune encephalomyelitis (EAE), animal models

## Abstract

Recently, there has been a substantial increase in the number of studies focused upon connecting the gut microbiome with cases of central nervous system (CNS) autoimmunity. Multiple sclerosis (MS) is a neurodegenerative autoimmune disorder of the CNS. Recent experimental and clinical evidence suggests the presence of microbial imbalances in the gut of MS sufferers. The gut microbiome is defined as the summation of all the microbial entities as well as their genes, proteins, and metabolic products in a given space and time. Studies show the MS gut microbiome as having general alterations in specific taxa, some associated with the promotion of inflammatory cytokines and overall inflammation. In conjunction with these findings, experimental models of the disease have reported that T regulatory (Treg) cells have deficits in their function as a result of the aberrant gut microbiota composition. The findings suggest that the interactions between the host and the microbiota are reciprocal, although more extensive work is required to confirm this. Moreover, evidence indicates that changes in microbiota composition may result in imbalances that could result in disease, with the gut as a potential novel therapeutic avenue. By understanding the biological effects of aberrant gut microbiome composition, it is possible to contemplate current therapeutic options and their efficacy. Ultimately, more research is necessary in this field, but targeting the gut microbiota may lead to the development of novel therapeutic strategies.

## 1. Introduction

Microbes are everywhere. Among them, bacteria are prokaryotic microorganisms with a vast array of functions and characteristics. Diverse bacterial metabolism allows them to occupy almost every possible niche on Earth. They can be found in conditions ranging from the extreme, such as in deep-sea hydrothermal vents, to milder conditions such as on plants growing in temperate climates. As a consequence, every facet of human biology is exposed to bacteria. Whether it is from the dust in the air or the food consumed, humans are constantly exposed to bacteria.

Generally, there is a misconception that bacteria are harmful to humans and pose a threat to human health. There are more bacteria that establish symbiotic relationships with us (commensal or mutualistic) than microbes that are pathogenic. The symbioses microbes participate in with their human host are analogous to symbioses present in traditional ecological settings. Commensal microbes will reside in or on the human host and receive nutrients from their host’s diet and or metabolites produced from other bacteria. These microbes do not benefit their host, but they are not detrimental either. Mutualistic microbes will behave much like commensals, but their presence will provide a direct benefit to their host. As science continues to investigate the bacteria associated with humans, their apparent function and benefits become more apparent.

Bacteria and other microbes must compete and interact with others and the surrounding environment. These interactions form intricate microbiomes; a microbiome is best defined as the summation of all these microbes as well as the combination of all the genetic material from them in a defined space and time. The sum of all microbes within a given niche is defined as microbiota. The microbiomes are generally defined by the physical and physiological space in which they are located. Particular interest has been focused on human-associated microbiomes due to their relevance to human health. Because of this, and in association with its highly complex structure and broad array of effects, the best studied microbiome is the human gut microbiome (and by proxy the murine gut microbiome).

Gut microbes have significant impacts on metabolism and immune and neuronal responses. As a result, the microbiota can potentially affect the onset and progression of diseases defined by several effector cells and soluble metabolic, immune, and neuroendocrine factors modulated by gut microbes. For instance, experimental evidence indicates that gut microbes affect the balance of pro- and anti-inflammatory immune cell populations and cytokines linked pathologically with different autoimmune diseases. Furthermore, an increasing number of studies now report on differences in the percentages of specific microbial taxa found in the gut content of patients that suffer from autoimmune diseases such as multiple sclerosis (MS), and healthy individuals. In this review article we discuss the proposed association established between the gut microbiota and MS.

## 2. The Gut Microbiome

The best studied microbiomes are the mouse and human gut microbiomes that span the entire digestive tracts, including the stomach, small intestine, caecum, large intestine, and rectum. Across geographic locations, the gut environment varies dramatically in both its physical and chemical nature. The physical and chemical variation dictate the structure of the microbiota present. For example, the approximate pH of the duodenum is 6, whereas the pH of the terminal ileum ranges from 6 to 7.4 [1]. Mucus levels tend to differ between the large and small intestines as well, directly regulating the bacterial communities present [2]. Although the physical properties of the gastrointestinal tract shape bacterial populations, microbes persist and thrive. Therefore, the gastrointestinal tract physiology has evolved to interact with and respond to the microbiota present.

Bacterial metabolism also plays a role in which taxa are present at differing biogeographic locations. For instance, fatty acids and simple carbohydrates derived from food are absorbed in the small intestine. There, fermenting bacteria process these complex carbohydrates [3]. *Bacteroides spp.* are the most studied taxa that can perform this metabolic task [4]. Additionally, human diet plays a critical role in shaping the gut microbiome. Experimental and human studies have shown marked differences in the composition of the gut microbiome of obese individuals [5,6,7]. Studies in which humans switch their diets from being primarily plant-based to being primarily animal-based experience a profound effect on the composition of the gut microbiota [8]. The effect of diet on the composition of the gut microbiome generally is centered on the fact that it dictates what nutrients are available for the microbiota. Therefore, nutrient availability as well as retention is critical. Controlled dietary studies have shown that the effects of diet on the composition of the microbiota occur within short periods of time. In the study performed by David et al., the effects of dietary changes on the human microbiome were also reflected in the concentration of key metabolites with demonstrated impact on the immune system (such as short chain fatty acids); this will be discussed more later [8].

Antimicrobials play an additional role at shaping the structure of the gut microbiome. Specialized epithelial immune cells known as Paneth cells secrete antimicrobial compounds that alter the growth of bacteria near the mucosal surface [9]. These compounds are cationic peptides that interact with charged membranes of bacteria. Some bacteria, however, have evolved to respond to these charged peptides; some gram-negative bacteria have modifications in the lipid A component of the outer membrane, which renders them resistant to these peptides [10]. Interestingly, however, the concentration of antimicrobials is higher towards the proximal end of the small intestine, which results in higher abundance and diversity in the distal ends [3].

Secreted immunoglobulin A (sIgA) and other immune system-mediated responses dictate what bacteria persist where. The intestinal mucosa contains large quantities of sIgA to monitor the gut microbiota [3]. The non-pathogenic bacteria become coated in sIgA to maintain tolerance from the host. sIgA coatings on bacteria reduce inflammatory signaling and reduce changes to bacterial gene expression [11]. This process allows homeostasis between the host and the microbiota to be maintained. However, this process can sometimes be utilized by pathogenic bacteria as well. In the case of some species of *Helicobacter*, these pathogens are coated in sIgA as well, resulting in an inappropriate tolerance response by the host [12]. Therefore, the homeostasis between non-pathogenic bacteria and the host is more complex than solely sIgA coating. The promotion of tolerance as a mechanism of self-survival has been proposed. This is shown the case of *Bacteroides fragilis*. The polysaccharide A component of the bacterial capsule on *B. fragilis* has been shown to exert anti-inflammatory properties by stimulating the secretion of interleukin (IL)-10 by regulatory T (Tregs) cells [13]. This process is seen in other bacteria as well, suggesting that the need for self-promoting tolerance is necessary.

### 2.1. The Anatomy of the Gut Epithelium

The gastrointestinal tract is the site of interaction between the body’s largest concentration of immune cells and the gut microbiota [14]. The gut epithelium acts as a major barrier between the external environment and the host’s internal environment. The human digestive tract essentially is a long tube starting from the mouth and extending down the esophagus, past the stomach, and through the small intestines and large intestine, finally ending past the colon at the anus; everything humans eat needs to be separated from the inside of the body.

Goblet cells in the intestinal epithelium produces mucus to form a matrix between the external environment and the intestinal epithelial surface [15]. Besides the mucus layer, only a single layer of epithelial cells separates the intestinal lumen contents from the underlying connective tissue and interior milieu [16]. Due to this, the intestinal epithelium developed specialized cells types to deal with the exposure. As previously discussed, Paneth cells play a critical role in maintaining the security of the gut epithelium by secreting antimicrobial peptides. Additionally, cells expressing *CD24* reside in the colonic crypts which elicit similar responses to the Paneth cells [17].

The thin layer of epithelium generates a barrier that can prevent material from the intestinal lumen from entering the interstitial space of the body. Tight junction protein complexes regulate the paracellular permeability of the intestinal epithelium [16]. The permeability of the epithelium varies across the intestines geography and is largely determined by the amount of tight junction protein complex expression. The protein complex consists of transmembrane proteins such as occludin, claudin, junctional adhesion molecules, tricellulin, and intracellular scaffold proteins like zonula occludens [16]. The pathogenesis of several diseases such as inflammatory bowel disease, celiac disease, and even food allergies have been attributed to the hyperpermeability of the intestinal barrier [18]. Interestingly, alterations in normal gut microbiota has been noted to cause changes in intestinal permeability [19]. In the case of Gulf War illness, chemical injury caused an alteration in intestinal microbiota, resulting in a down-regulation of occludin expression and an increase in intestinal permeability. The leachates then caused endotoxemia, leading to the upregulation of toll-like receptor 4 (TLR4) activation in the intestine as well as the brain [19].

Because of the high intake of microbes via digestion and the critical role intestinal epithelia plays, the immune system and intestinal physiology evolved to monitor the traffic. The lymphatic system is a network of vasculature that allows leukocytes to travel through the body and monitor antigens. For the gut, there is a specialized set of lymphatic tissue known as the gut-associated lymphoid tissue (GALT). The GALT comprises the Peyer’s patches, mesenteric lymph nodes, lymphatic vasculature, as well as other lymphoid aggregates. These structures work in tandem to protect the gut from pathogenic microbes while monitoring the commensal or mutualistic bacteria.

### 2.2. The Gut/Immune System Nexus

As bacteria and their metabolites persist in the gut, the GALT-associated immune cells must monitor every aspect of the intestinal lumen to catch and eliminate pathogens. The Microfold (M) cell, a specialized epithelial cell of mucosa-associated lymphoid tissue, transports antigens from the intestinal lumen to immune cell populations. M cells allow dendritic cells to sample antigens from the lumen and endogenously prepare the antigen for presentation to T effector cells via the major histocompatibility complex (MHC) class II molecule. This process then, in turn, can activate the T cells and differentiate them into specialized effectors to perform functions necessary for dealing with the microbe expressing the activating antigen. Whether it is to mount an immunological response or to disregard the antigen, an interaction between the host immune system and the microbe will occur. This process is mediated by the subsets of T cells as well as anergy.

Pro-inflammatory T cells are required for the control of pathogenic microbes. T helper (Th) cells such as Th1, Th2, and Th17 can help mobilize and recruit innate immune cells to the site of the pathogen. This process is mediated by the secretion of cytokines and chemokines. Cytokines are molecules such as interferons, interleukins, growth factors, and other compounds that have some effect on other immune cells. Cluster of differentiation (CD)4 T cells express surface receptors known as T cell receptors that can identify antigens presented by MHC molecules. Upon this union, the naïve T cell will differentiate into one of the various T helper cell subsets depending on the additional signaling molecules the naïve T cell encounters during co-stimulation. As an example, Th1 cells can be activated after co-stimulation and go on to secrete interferon-gamma (IFN-γ) to activate other Th1 cells in the immediate area [20]. The non-cognate stimulation of Th1 cells allows the immune system to clear pathogenic intracellular bacteria. These cytokines and chemokines recruit innate immune cells such as neutrophils to the site of the infection; the immune system then can eliminate the threat of the pathogen via phagocytosis, neutralizing antibodies, or antibody mediated engulfment.

Tolerogenic responses are by contrast required to maintain a sustained population of symbionts. As previously stated, not all bacteria are pathogenic but rather commensal or mutualistic. Therefore, it is critical for the immune system to have a way to virtually ignore the antigens of these non-harmful bacteria. As previously discussed, *B. fragilis* had the ability to stimulate its own immunotolerance by stimulating the production of Tregs which would secrete IL-10 [10]. In addition, anergy could also be used to prevent an immunological response against symbionts. Anergy is defined as being the absence of a normal immunological response to an antigen; achieving anergy against non-pathogens could be another way to ignore non-pathogenic antigens. Endosomal TLR7 can be engaged resulting in an intracellular calcium flux with the activation of a NFATc2-dependent anergic gene expression program that ultimately results in T cell non-responsiveness [21].

The processes that maintain the balance between pro- and anti-inflammatory processes are complicated and, in some cases, depend on the bacteria or viruses present [21]. Additionally, the gut microbiome is shaped by various factors including host genetics, geographical location, diet, lifestyle choices, prescribed pharmaceuticals, mode of delivery during birth, antibiotic exposure, and possibly even disease states themselves. When these factors tip the gut microbiome out of balance, there can be an induced imbalance between these pro- and anti-inflammatory responses that can result in disease. This proposed disease model is known as dysbiosis: gut microbial imbalances that result in, or are a result of, disease states. In general, laboratories are investigating whether these alterations in gut microbiota result in disease. However, it is possible that alterations in the balance between pro- and anti-inflammation can alter the structure and function of the gut microbiota as a function of diseased states.

## 3. Multiple Sclerosis and Autoimmunity

Autoimmunity is best described as being a process in which the host’s immune system fails to distinguish the self from non-self and begins to elicit immunological responses against host tissue. Mechanisms that drive autoimmunity remain to be elucidated; however, it generally is understood that exacerbated pro-inflammatory responses can exacerbate the tissue damage that characterizes the process of autoimmunity. Therefore, understanding the extent of inflammation induced by dysbiosis becomes critical. The importance of gut microbiota in context to immune function can be seen when comparing traditional mice to gnotobiotic mice, also referred to as germ-free (GF) mice. GF mice exhibit reduced immune function with physiological abnormalities such as increased intestinal permeability. Dysbiosis can be induced by the same environmental factors that have been described to potentially illicit autoimmunity. Moreover, dysbiosis has been noted in several experimental models of autoimmunity as well as in patients suffering from autoimmunity [22,23,24,25]. These phenomena can provide evidence that intestinal dysbiosis is critical in the development of autoimmune disorders.

Multiple sclerosis (MS), a clinically common autoimmune disease, affects millions of individuals worldwide; many of these individuals reside in northern geographic locations. For these individuals the quality of life slowly diminishes as the disease progresses; the host immune system attacks the insulating structure known as the myelin sheath that surrounds the axon shaft of neurons in the spinal cord [26]. When the sheath is degraded, the ability for electrical signals to be sent through the axon is reduced, thus resulting in paralysis and other symptoms. The most prevalent form of MS is relapsing–remitting MS (RRMS) that affects 85% of the total patient population; RRMS is initially diagnosed as a syndrome of neuronal dysfunction with a repeating series of relapses and remissions that follow over time. Approximately 70% of RRMS patients develop secondary-progressive MS (SPMS), which causes a steady and progressive neurological impairment [27,28]. The precise reason as to why immune cells destroy myelin remains unclear [27,28]. In MS, the myelin sheath that surrounds the axons of neuronal cells is degraded by host immune cell populations. Although the exact etiology is debated, there are hallmark events that undeniably occur.

Genetic variation is attributed to about one-third of the disease risk [29]. Environmental conditions as well as lifestyle choices play an additional factor in disease risk. Lacking a predominant exogenous risk factor, there is ambiguity as to whether MS starts in the periphery or in the central nervous system (CNS) [26]. In peripheral models of MS, pathogenic T cells are activated and subsequently released to the draining lymph nodes [26]. From the draining lymph nodes, these pathogenic T cells can enter circulation and gain access to the central nervous system by trafficking with activated B cells and monocytes [26]. Intrinsic models propose that the rise of autoreactive lymphocytes is secondary to intrinsic CNS damage [26]. Additionally, autoreactive B cells can be found in the meninges, parenchyma, and cerebrospinal fluid [26]. These autoreactive B cells can secrete antibodies which tend to increase with age in MS patients [30]. There might be unknown autoantigens, making the mechanisms behind autoreactive B cell pathology speculative [26]; however, next-generation sequencing has provided evidence that antigen-experienced B cells potentially go through maturation prior to entering the CNS [31]. Inflammation is a primary result from autoreactive lymphocytes. These responses cause axonal damage and could potentially trigger a self-sustaining chronic neurodegenerative process [26]. As a result, resident CNS cells such as the microglia and astrocytes additionally secrete inflammatory molecules, further exacerbating neurodegeneration [32]. The extent to which all these cell populations work in tandem to cause disease highlights the need to prevent the initial onset of inflammation and neurodegeneration.

Compounding the magnitude of autoreactive immune cell populations, defective Treg cells have also been noted in MS [33]. Defective Tregs could contribute to the production of autoreactive lymphocytes and additionally exacerbate the effects of preexisting autoreactive lymphocytes. Studies show that Tregs are lower in numbers and have reduced functionality in patients with MS [34]. Ultimately, it is the occurrence of defective Treg cells that can help explain why autoreactive immune cells arise. The degradation of the myelin sheath by host immune cells may be mediated by the T helper 17 (Th17) cells [35]. The imbalance between effector T cell and Tregs leads to the pro-inflammatory states which characterize MS. The increased levels of Th17 cells secrete pro-inflammatory cytokines and chemokines that recruit immune cells for the degradation events. Reduced and dysfunctional Tregs will fail to keep the exacerbated Th17 in check resulting in myelin degradation.

Gut microbial imbalances tend to shift towards a pro-inflammatory state that have profound effects on the intestinal physiology of the individual. Additionally, dysbiosis has been associated with intestinal barrier disruptions. When the integrity of these tight junction protein complexes diminishes there is an increase in intestinal permeability; the bacterial antigens can pass out of the intestinal lumen and travel to other locations in the body. As a result, levels of antigens, like the endotoxin lipopolysaccharide, can increase in the blood circulation which could have systemic inflammatory effects [36]. Systemic translocation of bacterial antigens can have a profound effect on CNS immunity and impact the integrity of the blood–brain barrier [37]. This process can result in the ultimate passage of autoreactive lymphocytes into the CNS and have direct access to the myelin sheath.

### 3.1. The Gut Microbiome and Multiple Sclerosis

**Figure 1:**
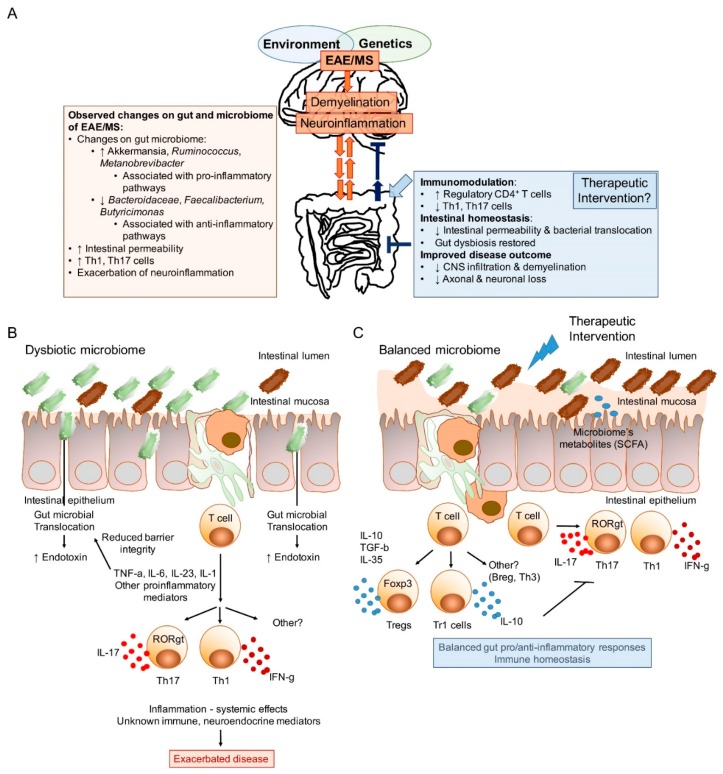
The bidirectional nature of the gut/brain axis, and the potential of the gut microbiome as a therapeutic route. (**A**) Proposed model of the gut microbiota as an amplifier of immune/inflammatory response. The exact genetic and environmental impacts on gut microbial composition is yet to be fully understood. Furthermore, the cellular and or soluble mechanisms of the reciprocal interactions between the microbiome and the host are still needed to be elucidated. Recent findings show significant alterations in microbial taxa of the gut microbiome and potential associations with pro-inflammatory pathways that can lead to or amplify disease. An effective therapeutic intervention targeting the microbiota might have to counteract dysbiosis, leaky gut that promotes microbial translocation and subsequent inflammation, thus modulating disease pathology. (**B**) Animal models suggest that autoimmune diseases including MS could be associated with gut dysbiosis, increased intestinal permeability, microbial translocation, and local and systemic inflammation. Inflammatory mediators, such as TNF-α, are known to reduce the expression of tight junction proteins thereby increasing the intestinal barrier permeability. (**C**) Experimental evidence obtained in animal models also suggest that interventions shifting the immune responses towards immunoregulatory pathways based on regulatory cells producing anti-inflammatory cytokines such as IL-10, TGF-β or IL-35 can restore immune homeostasis and protect against CNS inflammatory demyelination. Reduced inflammatory mediators as well as the direct production of metabolites such as SCFA by the balanced microbiome would facilitate increasing the compromised epithelial integrity. CSFA: short-chain fatty acids; CNS: central nervous system; MS: multiple sclerosis; EAE: experimental autoimmune encephalomyelitis; CD: cluster of differentiation; Th: T helper; IL: interleukin; TNF: tumor necrosis factor; TGF: transforming growth factor.

As previously discussed, autoimmunity has been shown to be impacted by the gut microbiome. However, it has also been theorized that disease itself can shape the structure and function of the gut microbiome. What this implies is that there is a bi-directional relationship between diseased states and the structure and function of the gut microbiome. This then raises more questions: What comes first, the disease or the aberrant gut microbiome? The proposed multifactorial and multidirectional association between the gut microbiome and the CNS of MS is described in Figure 1.

In some instances of autoimmunity, the aberrant gut microbiome precedes the onset of disease. There is an increasing interest in determining how disease itself shapes the gut microbiome. Risk factors that have been associated with autoimmunity also impact the gut microbiome [38]. Additionally, autoimmunity can directly impact how the immune system responds to the gut microbiota. In the case of inflammatory bowel diseases, the immune system targets resident microbiota, thus altering the overall structure of the gut microbiome [39]. Targeting non-pathobionts and clearing them from the intestines could have profound impacts on the immune system of the host. If a population of bacteria that promotes anti-inflammatory responses is eliminated it is possible that unchecked systemic inflammation could occur, thus further exacerbating the initial autoimmune disease.

The hypothesis that the gut microbiome is an environmental modulator of CNS inflammatory demyelination was first tested in experimental autoimmune encephalomyelitis (EAE), the most widely used animal model to study MS. The treatment with a broad-spectrum antibiotic intervention affected the balance between inflammation and inflammatory regulation in EAE by modulating intestinal microbiota and, as a consequence, Treg cell populations [40]. Additionally, Yokote et al. reported similar findings but noted that the alterations in intestinal microbiota impacted the natural killer T (NKT) cell populations [41]. These experiments analyzed the impacts antibiotic intervention could have on modulating EAE in SJL as well as C57 mice. Utilizing antibiotics to therapeutically target the gut microbiome has been proposed for models of diabetes [42] as well as ulcerative colitis [43]. A study conducted by Nakamura et al. set out to test the efficacy of an antibiotic cocktail on an experimental autoimmune uveitis (EAU) model. Autoimmune uveitis has both a genetic and environmental culmination that impacts disease susceptibility and is characterized by a distinct increase in Th17 cell populations with a decrease in Treg cell populations [44]. Utilizing an antibiotic cocktail consisting of ampicillin, vancomycin, neomycin, and metronidazole, the researchers noted that clinical scores of EAU were significantly reduced compared to the control group when the antibiotics were administered orally. The bacterial phyla Firmicutes and Bacteroidetes as well as the class of Alphaproteobacteria were reduced, while there was an increase in the class of Gammaproteobacteria. More importantly, the utilization of antibiotics significantly increased the expression of Foxp3^+^ Treg cell populations with a reduction in IL-17 producing Th17 cells [44]. Taking this all into consideration, the treatment of broad spectrum antibiotics conferred protection against the EAU pathology.

More recently, a study performed in non-obese diabetic mice showed that early treatment of disease with antibiotics delayed the onset of EAE, reduced severity and the progression of disease. The protection was observed when mice were treated between days 0 and 14. The treatment of EAE mice with same antibiotics at later days (30–44 and 70–84) did not affect disease progression and severity [45]. In this later study, we hypothesized that the interaction between the gut microbiome and CNS disease is bidirectional. We compared the gut microbiota composition of non-obese diabetic (NOD) EAE mice on days 0, 14, 30 and 58. We compared the gut microbiome of NOD mice induced with EAE. In our study, approximately 70% of mice exhibited disease progression and developed a severe form of EAE. When averaging the clinical scores of the remaining mice, the resulting pattern showed a continuation of mild disease throughout the duration of the experiment. We found that the mice which developed a severe secondary form of EAE harbored a dysbiotic gut microbiome when compared to the healthy control mice, and that the differences were observed at early stages of disease [45]. It might be relevant to note that only early intervention with antibiotics affected the progression of disease, while only early stages of disease showed effects with respect to the composition of the microbiota. The recent findings suggest then that the interaction between the gut microbiota and neuroinflammation is reciprocal. However, the experimental evidence summarized later in this review strongly indicates that changes in the composition of the gut microbiota significantly affect neuroinflammation and the disease course, which opens new therapeutic avenues to explore.

The effects of the lack of microbiota on health and disease have been explored using experimental GF animals. GF mice colonies are generated and maintained under constant sterile conditions. Significant anatomical and immunological effects result from the lack of exposure to microbes and microbial antigens mice: these mice show reduced numbers Peyer’s patches and lymphoid follicles in the gut associated-lymphoid tissues. These secondary lymphoid tissues are also smaller and harbor a reduced number of T cells than conventional, specific pathogen-free (SPF) mice. In the context of immune function these animals show biased responses characterized by reduced frequencies of pro-inflammatory Th17 cell subsets [46]. The altered immune system of GF mice influences their susceptibility to experimental autoimmune diseases. GF mice are less susceptible to glucose intolerance than mice housed conventionally [47]. Reduced susceptibility to disease has also been observed in models of inflammatory bowel disease [48], rheumatoid arthritis (RA) [49], and spontaneous [50] and actively induced EAE [51], among others. It is important to note, however, that same gut-associated alterations constitute a barrier for appropriate development of the immune system and brain function, which could be considered by many an experimental limitation for models designed to study complex, multifactorial diseases such as multiple sclerosis.

Significant changes in specific microbial taxa have been observed in the intestinal microbiome of MS patients when compared to healthy controls [52,53,54], evidence that might support the hypothesis that the gut microbiome can play a role in the development of MS. However, very little is known whether the disease affects the composition of the gut microbiome. Understanding how MS pathology affects gut microbiota can give insight to novel therapeutic approaches to impact disease progression. Similarly, dysbiosis drives disease progression in the inflammatory bowel disease model, therefore it is possible that dysbiosis also promotes inflammation in the MS model. 

The effects of the gut microbiota on other neurological diseases are also being extensively evaluated, as evidenced by the dramatic increase observed in the number of published works in the recent years [55]. Changes in the gut microbiota composition have also been observed in patients suffering from diseases such as neuromyelitis optica [56] and Parkinson’s disease [57,58,59]. Experimental models of neurological diseases such as autism spectrum disorders [60,61,62] and behavioral disorders [63,64,65,66,67] further suggest the influence of the gut microbiome on these pathologies. Other neurological diseases are also being currently evaluated in the context of the microbiome, such as Alzheimer’s disease, Huntington’s disease, and amyotrophic lateral sclerosis [55].

It is hypothesized that there is a bi-directional relationship between the gut microbiota and MS [68]. The composition of the gut microbiome might shape MS pathology at the same time MS disease progression also could alter the gut microbiome. However, two recent works clearly suggest that the changes in microbiota composition drive neuroinflammatory effects rather than the opposite. These recent studies support the premise that changes observed in the gut microbiome of MS patients are correlated with functional mechanisms that might regulate disease. Fecal transplantation of the gut microbiome can influence the progression of EAE. When fecal material from discordant monozygotic twins was transplanted into mice, there was a profound impact on spontaneous EAE disease incidence [69]. The MS gut microbiome had the ability to increase the likelihood of spontaneous EAE induction in mice as opposed to the healthy twin. Moreover, the stool from MS patients also increased the severity of EAE in mice [70]. These recent works clearly support the proposed concept that although interactions may be bidirectional the composition of the gut microbiota affect the progression of CNS inflammatory demyelination. Therefore, it is clear that targeting the gut microbiome might have profound impacts on MS pathology. Table 1 summarizes the colonization studies transferring MS microbiota into GF mice, as well as approaches based on monocolonization, multi-species colonization, and the treatment with immunomodulatory compounds purified from gut symbionts, discussed next. 

### 3.2. Targeting the Gut Microbiome with Therapeutic Options

As summarized earlier, recent experimental evidence and clinical data suggests that the gut microbiome might be a major factor regulating autoimmunity. Targeting the gut microbiome with therapeutics could have profound effects in disease progression as well as managing symptoms of disease. The extent and approach towards modulating the gut microbiome can follow many directions; it is crucial to balance the positive and negative impacts of each therapeutic option. Gut microbiome-based therapeutic approaches could have beneficial effects in terms of disease management but also have unintended adverse side effects. Furthermore, some therapeutic options can work for some autoimmune diseases but not others. Therefore, again, assessing proposed therapeutics are crucial and developing therapeutics with less negative consequences is essential.

#### 3.2.1. Antibiotic Therapy

As discussed earlier, EAE, a model disease for MS, has been explored in the context of antibiotic intervention [40,41]. Other CNS diseases also have studied in terms of how antibiotics impact disease pathology. Parkinson’s disease (PD) patients report gastrointestinal distress as well as exhibit intestinal inflammation well before symptoms of motor deficits [71,72,73]. A study hypothesized that alterations in the gut microbiota by antibiotic interventions could alleviate PD pathology due to the association of intestinal complications and the tight interactions between the gut and the CNS [74]. The treatment of broad-spectrum antibiotics did, in fact, confer protection against motor dysfunction in a murine model. These findings support the notion that therapeutic targeting of the gut microbiome with antibiotics may be efficacious.

The usage of broad spectrum antibiotics can also have adverse effects by the same mechanism. *Clostridium difficile*, an opportunistic pathogen, can infect the host after antibiotic treatments with symptoms ranging from diarrhea to pseudomembranous colitis and can be in some cases life threatening [75]. The exposure to antibiotics causes structural changes in the gut microbiome, leaving the host susceptible to opportunistic infection from *C. difficile* as well as other enteric pathogens [76]. Children exposed to antibiotics within the first three years of life exhibit lower diversity in their gut microbiome. Moreover, these microbiome structures are less stable in exposed children than in non-exposed children [77].

#### 3.2.2. Phage Therapy

In response to the negative impacts of broad spectrum antibiotic usage the notion of using bacteriophage (phage) as a therapeutic approach has been discussed. Phage are bacteria-specific viruses that can be altered to target bacterial populations of interest while leaving others untouched. The oral delivery of phage has been considered to be safe and have the capability to bypass intestinal epithelia and penetrate the GALT as well as the blood stream [78,79]. This ability to penetrate the GALT as well as the blood stream makes phage therapy extremely attractive to deal with bacterial translocation that can lead to diseased states.

Phages play a critical role in shaping the gut microbiome naturally and constitute the bulk of the intestinal viriome [80,81]. However, phages can also be pathogenic by contributing to intestinal dysbiosis; their uncontrolled destruction of beneficial bacteria can have an impact on the overall structure and function of the gut microbiome [82]. Additionally, phages also can have the unintended impact of horizontally transferring antibiotic resistance to their bacterial hosts [83]. Lysogenic phage have had antibiotic resistance genes associated within their genomes [84]. To some, this has rendered the notion of phage therapy obsolete. However, researchers have been designing “smart” phage cocktails that bypass horizontal gene transfer and appear promising [85]. Ultimately, there is still a lot of unanswered questions and concerns with regard to the safety and efficacy of phage therapies in terms of unintentional microbiome impacts.

#### 3.2.3. Fecal Microbiota Transplantation

The approach of antibiotic interventions and phage therapeutics is to target bacterial populations and remove or reduce them from the gut microbiome. Fecal microbiota transplantation (FMT) acts as whole gut microbiome replacements in hope of correcting aberrant gut microbiome structures and functions. The efficacy of FMTs is also quite high; thus, fecal transplantations is a common therapeutic to treat patients with *C. difficile* [86,87]. Conceptually, by eliminating the host’s aberrant gut microbiome and replacing it with a healthy gut microbiome, the diseased state will be rectified. The efficacy of FMT has promise in other diseases, including autoimmunity, such as inflammatory bowel disease (IBD) [88], a disease proposed to be associated with gut dysbiosis [89], and neurological disorders, as well. In IBD, a meta-analysis showed that FMT therapy put 45% of patients into clinical remission and reduced the need for other forms of anti-inflammatory therapeutics [90]. Symptom rescue was attributed to microbiota manipulation; the gut microbiome changes in the patients in response to microbiota transplant therapy [91].

To date, few studies have evaluated the impact of FMT on MS. One initial abstract described the positive effects of FMT on neurological deficits of three MS patients [92]. A recent case report work offered the first evidence of the effects of FMT in a patient suffering from secondary progressive MS (SPMS) [93]. In this report, a 61-year-old SPMS patient who had suffered from several episodes of enterocolitis triggered by *Clostridium difficile* in the past received FMT in 2006. Following the next 10 years, investigators monitored her Expanded Disability Status Scale (EDSS). The study showed that EDSS was stabilized suggesting the potential of FMT providing long-term benefit to the patients [93]. However, as the authors of the study discuss, although EDSS was stabilized, her symptoms did not improve suggesting a limited effect of the treatment. Furthermore, no records exist of the patient’s microbiome composition prior *C. difficile* infection thus it is difficult to speculate about the impact of the transplantation on MS. Further, large-scale studies are necessary to elucidate the potential therapeutic impact of FMT on MS.

There are still concerns regarding safety that remain to be addressed. Some of these concerns regard the route of administration, frequency of applications, screening the microbiota from the donor, the preparation protocol of the stool sample from the donor, what antibiotics should be administered and their frequency prior to fecal transplantation, among others [89]. Some of these concerns have been partially explored. For example, instances of aspiration occurred in two patients when the FMT was administered via gastroscopy [87]. It was proposed that that colonoscopy would be a safer route for the therapy. This claim was then assessed later in the study by Bamba et al. [86]. Still, several concerns remain. A systematic review of 50 reports on FMT indicated 28.5% reported cases of adverse effects, ranging from fever to nausea, abdominal cramps, dizziness, although there was a reduced frequency (2%) of other severe adverse effects [94]. Furthermore, the donor stool could significantly affect the intestinal homeostasis of the receiver. Studies of the composition of the gut microbiome and its impact on human health are still on their infancy and the potential implication of the transfer of significant numbers of microbes to a new host in metabolic, immune and neuroendocrine systems require attention. Ultimately, the risks and rewards of fecal transplantation therapy still need extensive exploration.

#### 3.2.4. Dietary Supplementation

Diet is the main factor shaping the structure of the gut microbiome. By manipulating compounds and nutrients the gut is exposed to, it is possible to alter the structure and function of the gut microbiome. Western diets generally consist of high amounts of saturated fats and carbohydrates which can lead to chronic inflammatory states [95]. Recognizing the impacts of the Western diet and identifying routes to supplement the diet to reduce the negative impacts and chronic inflammatory states could be a therapeutic avenue to assess. As was discussed earlier, studies in animal models and studies of the composition of the human gut microbiome in response to diet have demonstrated the drastic impact of changes of diet, which also occur within few days of dietary intervention [5,6,7,8]. The analysis of the human microbiome before and after plant vs. animal-based diets evidence the fast and significant effects that diet has on the microbiome composition [8]. The effects were also observed in the abundance of short-chain fatty acids (SCFAs). Acetate, propionate, and butyrate are SCFAs derived from the catabolism of carbohydrates. These SCFAs have shown to regulate a number of relevant molecular and cellular pathways associated with immunomodulation, such as the induction of Tregs [96], as well as the regulation of the permeability of the blood–brain barrier [97]. Thus, dietary factors have been shown to regulate the balance between pro- and anti-inflammatory responses that in turn might regulate autoimmune diseases, such as MS [98].

Naturally occurring compounds have been noted to have profound impacts on metabolic health. One of the most extensively evaluated in the context of MS is vitamin D [99]. MS patients show reduced levels of vitamin D that might be attributed to reduced sunlight exposure in high geographical latitudes but as recently discussed also to other factors independent of location, including the expression levels of vitamin D receptors, the host’s metabolism and the effects of the gut microbiota [98]. Due to the reduced levels of vitamin D observed in MS patients and its importance regulating the immune system [99], reducing intestinal permeability [100] and affecting the production of immunomodulatory metabolites such as butyrate [101], vitamin D supplementation is a potential therapeutic approach to treat the disease. However, the appropriate dosage of administration as well as the combination with other supplements remains to be elucidated. A combined therapeutic approach between vitamin D supplementation and other disease-modifying drugs has been also considered [102].

Utilizing dietary supplements as a therapeutic avenue for MS is still largely unexplored. The usage of probiotics however, has been gaining attention. A probiotic is generally described as bacteria that is administered orally to have some beneficial impact on the health of the individual. Moreover, probiotics are non-toxic immunomodulatory agents that could also be used orally in conjunction with current therapeutics for MS [103]. Studies have shown that oral administration of probiotics have positive immunomodulatory effects that could work in tandem with current pharmaceutical therapeutics.

#### 3.2.5. Probiotics and Immunomodulatory Factors Derived from Gut Microbes

GF models of EAE have been explored to determine the effects of individual microbial species of the microbiome on disease severity and progression. The reduced susceptibility of GF mice to spontaneous and induced EAE [50,51] was used as a baseline to compare the effects of the mono-colonization with *segmented filamentous bacteria* (SFB). SFB is a gram-positive bacterium that promotes the expansion of Th17 cells [104]. When GF EAE mice were reconstituted orally with SFB, the severity of EAE was also restored [51] (Table 1).

Other microbes have the ability to exacerbate EAE severity. The oral microbiome member *Porphyromonas gingivalis* exacerbates gliosis and inflammation that results in enhanced severity of EAE [105,106]. By contrast, other microbes have been shown to reduce the severity of EAE in mice and rats. *Bifidobacterium animalis* reduces EAE severity in Lewis rats [107], a mixture of *Lactobacillus* spp. reduces EAE severity in mice by inducing IL-10-producing Tregs [108], and the treatment with a genetically engineered *Lactococcus lactis* designed to express the heat shock protein 65 (hsp65) also promotes EAE protection by inducing Tregs [109]. IL-10-producing Tr1 cells have also been proposed as having a mechanism of action that drives EAE protection, promoted by *Pediococcus acidilactici*, another lactic acid bacterium [110]. More recently, the gut microbiome member *Prevotella histicola* has been identified as a potential immunomodulatory bacterium with enhanced ability to promote protection against inflammatory CNS demyelination by reducing Th1 and Th17 cells and enhancing Tregs and tolerogenic dendritic cells, as well as immunosuppressive macrophages [111]. Our previous work showed that the polysaccharide A (PSA) produced by *Bacteroides fragilis* promotes immunomodulatory responses that reduce the severity of EAE [112,113,114,115]. PSA in mice expands the populations of IL-10 producing regulatory CD4^+^ T cells, including Tregs, IL-10-producing T cells (defined as Tr-1 that do not express the transcription factor foxhead box P3 (Foxp3) that defines Tregs) [116,117,118], and CD39^+^ T cells [114,115] (Table 1). Interestingly, previous works by other authors showed that CD39^+^ Tregs are impaired in their ability to control the proliferation of IL-17-producing T cells isolated from patients suffering from MS [119]. The ectoenzyme CD39 (triphosphate diphosphohydrolase 1: ENTPD1) control the catalysis of ATP into AMP. The effects of PSA depend on its recognition through toll-like receptor 2 (TLR2) by conventional [114,116] and plasmacytoid dendritic cells [120].

The immunomodulatory effects of PSA have been demonstrated in cells derived from human peripheral blood mononuclear cells (PBMCs). In a first study, blood from healthy individuals was used to isolate naïve CD4^+^ T cells and dendritic cells for in vitro Treg induction assays. Exposure of PSA to human dendritic cells induced CD39^+^FoxP3^+^ Tregs [121]. Furthermore, circulating Foxp3^+^CD4^+^ T cells isolated from healthy individuals and cultured with PSA-exposed dendritic cells showed an enhanced expression level of CD39 and IL-10 production, and reduced tumor necrosis factor (TNF)-production by monocytes stimulated with lipopolysaccharide (LPS) [121]. In a more recent follow up study, PSA was shown to increase dramatically the production of IL-10 by CD4^+^ T cells isolated from patients suffering from MS [122]. In this later study, PSA was able to stimulate the expression of Foxp3 in CD4^+^ T cells sorted from PBMCs of healthy untreated MS patients, and MS under treatment with glatiramer acetate (GA) when compared to untreated cells. Similar increases were observed in the context of IL-10 produced by cells exposed to PSA when compared to untreated cells. Remarkably, the fold-increase in Foxp3 expression was higher in cells isolated from MS patients than in cells isolated from healthy individuals [122]. Thus, in vivo and in vitro studies have demonstrated that PSA, a polysaccharide produced and isolated from a gut microbiome member promotes immunomodulatory responses in EAE mice and in samples isolated from MS patients.

## 4. Concluding Remarks

All the potential new therapeutic options discussed in this review have not been explored fully for MS. Although there is still no cure for MS, over the last two decades the number of therapeutics available for MS has increased significantly. Some therapeutics available only treat symptoms of the disease and act as immunosuppressive or immunomodulatory agents; however, many of them have limited efficacy and have in some cases severe side effects [123]. Moreover, as of yet there is no efficacious therapeutic for secondary progressive MS. Because of the lack of a therapeutic that promotes efficacy to all patients with no associated side effects, there is a need to explore novel therapeutic avenues.

The effect of the gut microbiome on MS pathology is a promising avenue of investigation. Therefore, characterizing the microbial profile of the MS gut as compared to healthy gut microbiomes, as well as other autoimmune diseases could be insightful. By investigating the structure of these microbiomes, it is possible to understand what microbes are contributing to the pro-inflammatory state and lack of anti-inflammation. From this data, it would be possible to then develop probiotic mixtures containing critical bacterial populations which can increase the number of Tregs as they are diminished in the MS gut microbiome.

## Figures and Tables

**Table 1:** Experimental interventions of the gut microbiota that modulate CNS inflammatory demyelination.

Microbial Intervention	Model	Experimental Outcome	Proposed Mechanism of Action	Refs
**Colonization studies in germ-free (GF) animals or mice treated with antibiotics**				
Monocolonization of GF with segmented filamentous bacteria (SFB)	Mouse, EAE	Disease susceptibility restored	GF mice: Reduced peripheral Th17 cell and increased Treg frequencies and anti-inflammatory cytokines. Reconstitution with SFB-induced Th17 cells.	[51]
Monocolonization of GF with specific pathogen-free microbiota	Mouse (MOG-specific TCR Tg), EAE	Disease susceptibility restored	GF mice: Deficit in Th17 cell in lamina propria and Peyer’s patches. Lack of autoimmune T cells and B cell recruitment and autoantibody production reduced. SPF microbiota restores susceptibility	[50]
Antibiotics + PSA-producing *Bacteroides fragilis*	Mouse, EAE	Disease severity restored (PSA-production dependent)	Antibiotics treated: disease reduction [38,39]. PSA-deficient, but not PSA-producing *B.* *fragilis* restores EAE susceptibility by promoting IL-17-producing and interferon-gamma proinflammatory cells	[113]
Colonization of GF mice with gut microbiota of MS patients	Mouse, EAE	Restores disease susceptibility	MS gut microbiota reduces proportions and function of IL-10+ Tregs	[70]
Colonization of GF mice with gut microbiota of MS patients	Mouse (MOG-specific TCR Tg), EAE	Restores disease susceptibility	MS gut microbiota promotes Treg dysfunction and reduces immunoregulation by IL-10	[69]
**Colonization studies in conventionally housed animals**				
Colonization with *Lactococcus* spp.	Mouse, EAE	Reduced severity	Induction of IL-10-producing Tregs	[108]
Colonization with *Bifidobacterium animalis*	Lewis rats, EAE	Reduces EAE severity	Proposed changes in Th1/Th2 balances	[107]
Colonization with *Pediococcus acidilactici* R037	Mouse, EAE	Reduces EAE severity	Induction of IL-10-producing Tr1 cells	[110]
Colonization with *Lactococcus lactis* Hsp65	Mouse, EAE	Reduces the severity of EAE	Induction of Tregs cells and LAP^+^ CD4^+^ Tregs	[109]
Colonization with *Prevotella histicola*	HLA class II Tg mouse, EAE	Reduces the severity of EAE	Induction of Tregs, reduction of Th1 and Th17 cells function	[111]
**Treatment with purified symbiont factor isolated from gut microbiota**				
Oral treatment with purified PSA produced by *B. fragilis*	Mouse, EAE	Reduces the severity of EAE	Prophylactic and therapeutic protection by IL-10-producing CD39^+^ and Tregs	[113,114,115]

GF: germ-free; Tg: transgenic. MOG-specific TCR Tg: transgenic myelin oligodendrocyte glycoprotein (MOG)-specific T cell receptor. IL: interleukin; HLA: human leukocyte antigen. PSA: polysaccharide A.
